# Efficacy of Percutaneous Vertebroplasty Versus Placebo and Conservative Treatment in Osteoporotic Vertebral Fractures: An Updated Systematic Review and Meta-Analysis of Randomized Clinical Trials

**DOI:** 10.3390/diagnostics15212684

**Published:** 2025-10-23

**Authors:** Antonio Jesús Láinez Ramos-Bossini, Francisco Garrido Sanz, Marina Gea Becerra, Consolación Melguizo Alonso, José Prados, Fernando Ruiz Santiago, José Manuel Benítez

**Affiliations:** 1 Department of Radiology, Hospital Universitario Virgen de las Nieves, 18014 Granada, Spain; 2 Instituto de Investigación Biosanitaria de Granada (ibs.GRANADA), 18016 Granada, Spain; 3 Department of Human Anatomy and Embryology, School of Medicine, University of Granada, 18071 Granada, Spain; 4 Department of Radiology, Hospital Universitario San Cecilio, 18007 Granada, Spain; 5 Institute of Biopathology and Regenerative Medicine (IBIMER), University of Granada, 18100 Granada, Spain; 6 Center of Biomedical Research (CIBM), University of Granada, 18100 Granada, Spain; 7 Department of Computer Science and Artificial Intelligence, University of Granada, 18100 Granada, Spain

**Keywords:** percutaneous vertebroplasty, vertebral augmentation, placebo, conservative treatment, randomized clinical trial, pain, functionality, quality of life

## Abstract

**Introduction:** The efficacy of percutaneous vertebroplasty (PV) versus placebo and conservative treatment (CT) in patients with osteoporotic vertebral fractures (OVFs) has been debated in recent years. The aim of this study was to conduct an updated systematic review with a meta-analysis on the efficacy of randomized controlled trials (RCTs) comparing PV versus placebo and CT in pain relief, functionality and quality of life in patients with OVFs. **Methods:** A systematic search was conducted in PubMed, Web of Science, EMBASE, and CENTRAL, resulting in a total of 15 RCTs. The risk of bias was assessed using the Risk of Bias v.2 tool. A meta-analysis was performed using the weighted inverse variance method to analyze the standardized mean difference (SMD) in pain (VAS/NRS scales), functionality (RMDQ/ODI scales) and quality of life (QUALEFFO scale) in the short (<1 month), medium (1–6 months) and long terms (≥6 months). Heterogeneity was assessed using I^2^ and τ^2^. Subgroup analyses were performed according to the type of control, geographic region, number of institutions, fracture chronicity, and risk of bias. In addition, sensitivity (leave-one-out) and publication bias (funnel plots and Egger’s tests) analyses were performed. **Results:** Overall, PV showed benefits over the combined control groups in pain relief in the short (SMD: −0.68; 95%CI: −1.28–−0.07), medium (SMD: −0.63; 95%CI: −1.18–−0.07), and long terms (SMD: −0.59; 95%CI: −1.02–−0.15). No statistically significant differences were found in functionality and quality of life, although several trends toward significance were observed favoring PV. Subgroup analyses showed greater advantages of PV at several time intervals in acute (<8 weeks) OVFs, multicentric trials and studies with a low risk of bias. There were cues suggestive of potential publication bias in functionality, but not in pain or quality of life. **Conclusions:** PV shows significant benefits in pain relief, particularly in acute OVFs, but its efficacy in terms of functionality and quality of life remains unclear. These results support the use of PV in appropriately selected patients. However, given the high heterogeneity found, more controlled, multicenter trials are still required.

## 1. Introduction

Osteoporosis is the most common metabolic bone disease in the world, affecting more than 23 million people in Europe [1]. A recent meta-analysis estimated that the global prevalence of osteoporosis is close to 20%, with significant variability between countries, ranging from 4.1% in The Netherlands to 52% in Turkey [2]. In terms of gender and age distribution, there is a clear predominance in women over 50 years old (22–29%), while the prevalence in men at this age is around 6% [3,4]. This disease is characterized by a loss of bone quantity and quality, the main consequence of which is an increased risk of bone fractures. In Europe, approximately 4.3 million osteoporotic fractures are diagnosed every year, the most common site being the vertebrae (OVF). The economic burden of osteoporosis in this continent has been estimated at €56 billion [1], and these costs are expected to increase due to progressive population aging [5,6].

From a clinical point of view, the detection of an OVF implies the diagnosis of severe osteoporosis regardless of whether bone mineral density information is available, and requires immediate treatment due to the high risk of future fractures [7]. The most common location is the thoracolumbar spine, followed by the mid-thoracic segment [8], and the diagnosis is usually made using imaging tests, with magnetic resonance imaging being the most sensitive and specific, as it allows the presence of edema to be identified even in fractures without significant height loss [9,10].

Osteoporotic vertebral fractures are associated with significant morbidity due to pain, which often becomes chronic, and the resulting decrease in functionality and deterioration in quality of life, to which other potential alterations must be added, such as the occasional development of hyperkyphosis [11]. There is also an increase in mortality adjusted for sociodemographic characteristics and chronic comorbidities, with a relative risk of 1.22 higher than in patients without OVFs [12].

The approach to most OVFs, except those that cause neurological compromise (which is extremely rare), initially consists of analgesic measures and fixation of the fracture site using a brace [13]. This treatment is effective in a relatively low percentage of patients [14]. In cases of refractory pain, interventional approaches may offer benefits. These procedures are generally termed as percutaneous vertebral augmentation techniques, which include vertebroplasty, kyphoplasty, and vesselplasty, among others [15]. Percutaneous vertebroplasty (PV) is the oldest technique, first described in 1987 for the treatment of an aggressive vertebral hemangioma [16].

Briefly, PV consists of the introduction of biological cement through a system of needles percutaneously using real-time radiological guidance [17]. The objective is, on the one hand, to stabilize the fracture site and prevent progressive vertebral collapse, and on the other hand, to reduce pain. The pathophysiological process underlying pain relief has been attributed to neuromodulation of the adjacent sensory branches as a result of the exothermic energy produced when the cement solidifies [18], similar to what occurs in other approaches such as radiofrequency in lumbar facet joint pain [19].

Despite the widespread use of PV in clinical practice, its efficacy has been questioned [20], mainly as a result of a series of placebo-controlled clinical trials published since 2009 [21,22]. These publications did not find any benefits in favor of PV, which in turn led to the development of new clinical trials comparing both placebo and conservative treatment (CT). The most recent was published in 2023 by Carli et al. [23], and other clinical trials are currently underway. The results of these trials have been heterogeneous, both in terms of outcomes and methodological variables, including patient selection, placebo administration, follow-up time, and longitudinal evaluation, among others [24].

Far from having solidified the evidence for or against PV, there is currently an active debate about its usefulness. This has led to the development of numerous systematic reviews and meta-analyses [24,25,26] that attempt to clarify the available evidence and determine sources of heterogeneity that may explain the differences in the reported results. One of the most relevant is the Cochrane review by Buchbinder et al. (2018), which concluded that PV is not effective [27]. Since then, several meta-analyses that contradict this conclusion have been published, but most of them have highlighted the importance of introducing specific selection criteria for PV (e.g., [24,26]). The recent publication of the trial by Carli et al. (2023) [23] reactivates the existing controversy, as it showed overall beneficial results in favor of PV. However, no meta-analyses incorporating the results of this trial into the set of previously available trials have been published yet to our knowledge.

In sum, despite numerous trials and meta-analyses being to date, clinical uncertainty persists regarding the evidence supporting PV, as well as the optimal selection criteria to apply this technique in OVF patients. This lack of consensus continues to influence real-world decision-making, leading to variable clinical practices worldwide. Therefore, the present systematic review and meta-analysis aimed to clarify the current evidence by integrating the most recent randomized controlled trials (RCTs) to determine whether PV provides consistent advantages over placebo and CT in terms of pain relief, functional recovery, and quality of life.

## 2. Methods

### 2.1. Study Design and Eligibility Criteria

The systematic review and meta-analysis were conducted in accordance with the recommendations of the Preferred Reporting Items in Systematic Reviews and Meta-Analyses (PRISMA) guidelines [28]. The PRISMA checklist can be consulted in Supplementary File S1. The protocol for this study was registered in PROSPERO under registration code CRD420251057152. The research question was defined according to the PICO criteria. The study population (P) consisted of adult patients with OVFs; the intervention (I) was PV; the comparator (C) was CT or placebo; and the main outcome variables were pain, functionality, and quality of life.

The inclusion criteria for studies were published RCTs comparing PV with CT or placebo in OVF, and including quantitative results for at least one of the primary outcomes (pain, functionality, or quality of life) in both groups. Exclusion criteria were studies with vertebral fractures of non-osteoporotic etiology, and designs other than randomized clinical trials, as well as article types such as letters to the editor, abstracts, or conference proceedings.

### 2.2. Database Search Strategy

A search was conducted in the PubMed, EMBASE, Web of Science, and CENTRAL databases, using different combinations of the terms “vertebroplasty,” “conservative treatment,” and “placebo”. The search equations in each of the databases can be found in Appendix A.1, Appendix A.2, Appendix A.3 and Appendix A.4. To increase the sensitivity of the search, the references of relevant screened articles and previous systematic reviews on this topic (cluster search) were analyzed, as well as articles that subsequently cited the selected studies (snowballing search). No other sources such as gray literature or unpublished trials were investigated for feasibility reasons. The search was updated to 11 August 2025. No timeframe or language restrictions were established.

Based on the initial search, two authors (MGB and AJLRB) independently screened the results by reading the title and abstract. The filtered articles were then read carefully and the inclusion and exclusion criteria were applied. In case of doubt or discrepancy, a senior author (CMA) was consulted to reach consensus on the final decision to include or exclude the study. For articles not available in English or Spanish, the document was translated using the web version of DeepL Pro Translator tool (DeepL SE, Cologne, Germany) [29] to assess the inclusion of the study and, eventually, extract the data as described in Section 2.4.

### 2.3. Risk of Bias Assessment

The Risk of Bias Tool v. 2 (RoB2) [30], widely validated and used in previous systematic reviews and meta-analyses, was used to assess the risk of bias in the included trials. In summary, RoB2 includes five domains related to potential biases in a clinical trial (derived from the randomization process, deviations from the planned intervention, lack of data on outcomes, measurement of outcomes, and selection of reported results), which are assigned the category of “low risk”, “some concerns”, or “high risk”. The same researchers who performed article selection assigned the corresponding categories to each of the five domains in each study. In case of differences in the assigned category for each domain between both reviewers, the same senior author (CMA) was consulted to solve discrepancies.

### 2.4. Variables of Interest and Data Extraction

The two authors who selected the articles independently collected the main characteristics of the study in a spreadsheet created for this purpose, including sociodemographic variables of patients (age and gender distribution), country or countries where the studies were conducted, characteristics of the control group, duration of symptoms before the procedure, and follow-up time. The outcome variables were pain (measured using the visual analog scale [VAS] or numeric rating scale [NRS]), functionality (measured using the Oswestry Disability Index [ODI] and Roland-Morris Disability Questionnaire [RMDQ]), and quality of life (assessed using the Quality of Life Questionnaire of the European Foundation for Osteoporosis [QUALEFFO] osteoporosis-specific questionnaire, in any of its versions).

When disaggregated VAS information for a given trial was provided at rest or during forward bending, it was split as two independent studies [31]. In one of the studies analyzed [32], an error was identified in the QUALEFFO confidence interval (CI) at 12 months in the control group, established as 38.05-36.13 in the article for a mean value of 42.09. In this case, it was assumed that the upper limit corresponded to 46.13, in accordance with the mean value.

### 2.5. Management of Missing Data

Several of the included studies did not explicitly report the standard deviation (SD) value in the outcome variables throughout the follow-up, so it was estimated based on the available information, following Cochrane’s recommendations and previous studies, as follows:

1. When data were provided only in the form of graphical representations, PlotDigitizer [33] was used to obtain the mean, except when models used in the original trials did not allow direct extrapolation of the SD (e.g., Yang et al., 2016 [34]).
2. When data were reported as the difference in means from baseline, SDs were calculated using the formula:

$${SD}_{c}\;\approx \;\sqrt{{SD}^{2}+{SD}_{b}^{2}\;-\;2\;\cdot r\;\cdot \;SD\;\cdot {\;SD}_{b}\;}$$

where *SD_c_* is the *SD* of the change from baseline, *SD_b_* is the baseline *SD*, and *r* is the Pearson correlation coefficient between the baseline SD and the change SD, which was set at 0.5 following a conservative criterion [35].

3. When only the 95%CI of the mean value of the variable of interest at follow-up was provided, the corresponding SD was estimated from the standard error using the following formulas:

$$SE=\;\frac{U_{L\;}-L_{L}}{2\;\cdot \;t}\to \;SD=SE\;\cdot \sqrt{N}$$

where *SE* is the standard error, *U_L_* is the upper limit of the range, *L_L_* is the lower limit of the range, and *N* is the sample size of the group, while t is the value of Student’s t-distribution for *n* − 1 degrees of freedom. Given that the sample sizes of the studies in which this estimation method was applied ranged from a minimum of 36 patients to a maximum of 90 patients, the denominator “*2 · t*” was approximated to 4, which led to a maximum variation of 0.06, considered negligible in this study.

4. When the follow-up information was the mean and range (e.g., [36] < 70), the SD was estimated using the formula proposed by Hozo et al. [37] (included in the Cochrane guideline [35]), rounding the denominator value to 4:

$$SD=\;\frac{U_{L}-L_{L}}{4}$$

5. When neither the SD nor information allowing its estimation was provided (e.g., SE, range, and difference from baseline), as in the case of functionality measured with the RMDQ scale in Klazen et al. (2010) [38], the SD was estimated using the median SD of the corresponding group [24,35].

### 2.6. Statistical Analysis

A meta-analysis was performed using the weighted inverse variance method under a random effects model, as the heterogeneity in this context is well known [24,27]. The estimator of the main variables was the standardized mean difference (SMD) with its corresponding 95%CIs using the Hartung-Knapp adjustment. Notably, the latter tends to provide conservative CIs, which may lead to slight discrepancies between 95%CIs and *p*-values. In such cases, trends toward significance were interpreted based on whether the 95%CI included the null value or not. The analyses of the main variables were performed independently for the short term (<1 month), medium term (1–6 months), and long term (≥6 months).

In all cases, an analysis of heterogeneity was included using the I^2^ and τ^2^ statistics, considering for the first case that there was low, moderate, or high heterogeneity for the thresholds I^2^ < 40%, 40% < I^2^ < 75%, and I^2^ > 75%, respectively, as in previous meta-analyses [39,40]. Forest plots were generated to visualize the results.

In addition, subgroup analyses were performed based on the following variables:

- Control group: CT vs. placebo.
- Number of institutions involved: single-center vs. multi-center.
- Geographic region: Western vs. Eastern countries.
- Chronicity of the fracture: fractures diagnosed before 8 weeks of the procedure vs. 8 weeks or more. This cutoff was established based on previous meta-analyses [41] and recent studies supporting pain sensitization mechanisms in OVFs after this period [42].
- Risk of bias: high risk vs. low/uncertain risk (according to RoB2).

Subsequently, sensitivity analyses were performed for the outcome variables by sequentially excluding each included study in order to estimate its contribution to the overall effect estimator (leave-one-out analysis). Finally, publication bias was analyzed by generating and visually inspecting funnel plots and Egger’s tests [43].

When both RMDQ and ODI values were reported for the same study, the analyses were performed using the RMDQ value. In cases where it was explicitly stated that the results were reported as “intention-to-treat analysis,” the original sample sizes of each group were considered. Otherwise, the sample sizes of the group at each follow-up interval were considered, as reported in the tables or in the patient flow diagram in the study (per-protocol analysis).

All statistical analyses were performed using R software (version 4.5.0. for Windows) [44], with the “meta” and “metafor” packages [45]. To facilitate the visualization and interpretation of the results, the analyzed data were rounded to the first decimal place. The threshold for statistical significance was set at *p* < 0.05.

## 3. Results

### 3.1. Characteristics of the Included Studies

**Figure 1:**
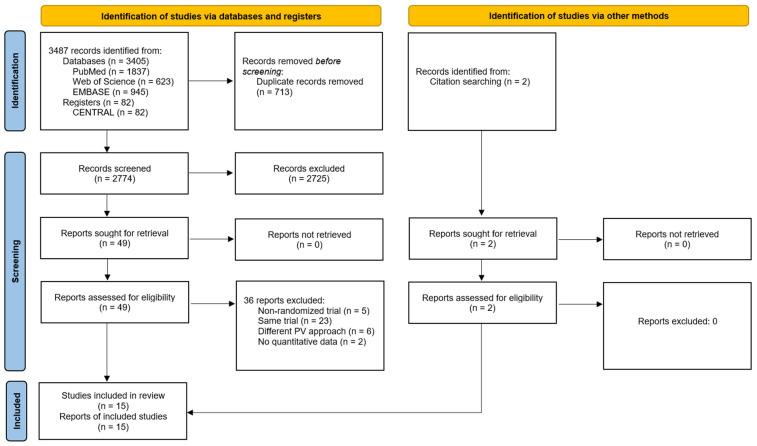
Flow diagram of the systematic review with meta-analysis performed according to PRISMA guidelines.

Following the article selection process, a total of 15 RCTs were included, as shown in the flow chart (Figure 1). The study by Hansen et al. (2016) [31], which included outcome measures assessed at rest and during forward bending (i.e., activity) was split into two separate studies, thus 16 different measures were considered in subsequent analyses. The studies excluded following full-text article reading can be consulted in Supplementary File S2.

The trials included encompassed a total of 1454 patients. Of these, 729 were in the PV group and 725 in the control group. Within the latter group, 312 were compared with placebo and 413 were compared with CT. Table 1 shows the main characteristics of the studies included in the meta-analysis.

### 3.2. Results of Risk of Bias Assessment

**Figure 2:**
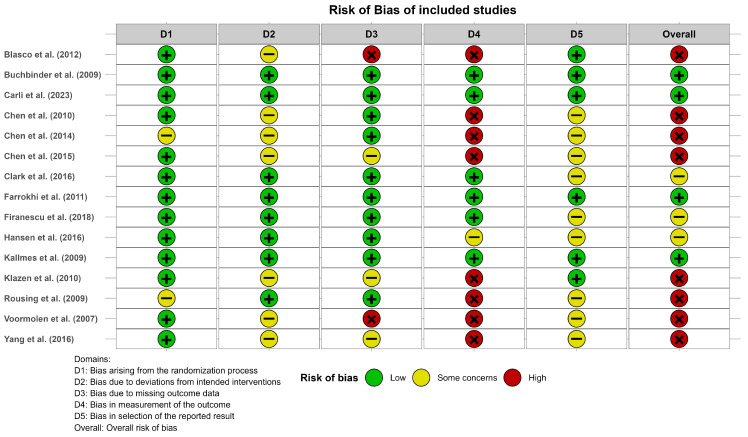
Risk of bias of the studies included in the meta-analysis according to the Risk of Bias Tool v.2. Blasco et al. (2012) [49], Buchbinder et al. (2009) [21], Carli et al. (2023) [23], Chen et al. (2010) [47], Chen et al. (2014) [50], Chen et al. (2015) [51], Clark et al. (2016) [52], Farrokhi et al. (2011) [48], Firanescu et al. (2018) [32], Hansen et al. (2016) [31], Kallmes et al. (2009) [22], Klazen et al. (2010) [38], Rousing et al. (2009) [46], Voormolen et al. (2007) [36], Yang et al. (2016) [34].

The risk of bias analysis using RoB2 showed that eight studies included at least one high-risk category (mainly in domain 4, due to bias in measurement of the outcome), three studies had at least one category of some concerns (mainly in domains 2 and 5), and four studies were considered as having a low risk of bias. Figure 2 shows the results of the risk of bias assessment. A complementary summary is provided in Supplementary File S3 and further details about domain-specific judgments can be found in Supplementary File S4.

### 3.3. Efficacy of Vertebroplasty in Pain Relief

**Figure 3:**
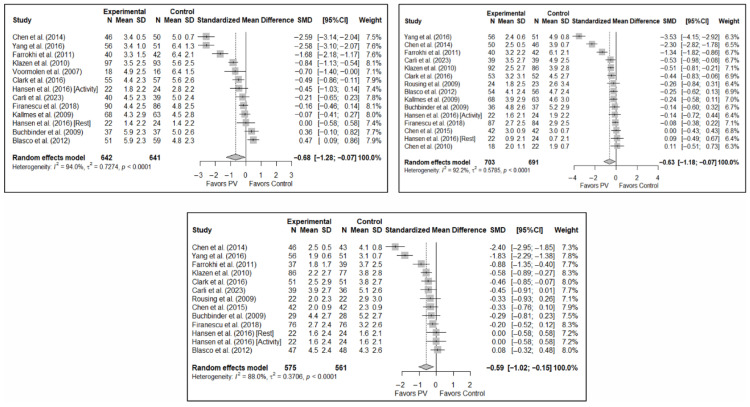
Forest plot of the analysis showing the efficacy of percutaneous vertebroplasty (PV) compared to the control group in short-term (**top left**), medium-term (**top right**), and long-term (**bottom**) pain. Overall *p*-values for short-, medium- and long-term analyses were 0.032, 0.029, and 0.013, respectively. Blasco et al. (2012) [49], Buchbinder et al. (2009) [21], Carli et al. (2023) [23], Chen et al. (2010) [47], Chen et al. (2014) [50], Clark et al. (2016) [52], Farrokhi et al. (2011) [48], Firanescu et al. (2018) [32], Hansen et al. (2016) [31], Kallmes et al. (2009) [22], Klazen et al. (2010) [38], Rousing et al. (2009) [46], Voormolen et al. (2007) [36], Yang et al. (2016) [34].

The results of the analysis of efficacy in pain relief revealed that PV showed statistically significant benefits compared to the control group in the short (SMD: −0.68; 95%CI: −1.28–−0.07), medium (SMD: −0.63; 95%CI: −1.18–−0.07), and long term (SMD: −0.59; 95%CI: −1.02–−0.15). Figure 3 shows the forest plot of the corresponding analysis.

### 3.4. Efficacy of Percutaneous Vertebroplasty in Functionality Improvement

**Figure 4:**
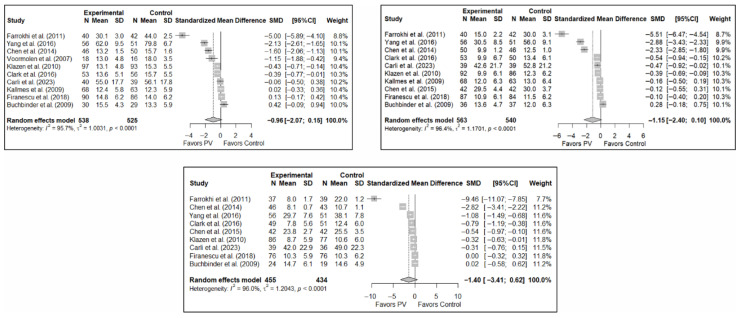
Forest plot of the analysis showing the efficacy of percutaneous vertebroplasty (PV) compared to the control group in short-term (**top left**), medium-term (**top right**), and long-term (**bottom**) functionality. Overall *p*-values for short-, medium- and long-term analyses were 0.083, 0.068, and 0.149, respectively. Buchbinder et al. (2009) [21], Carli et al. (2023) [23], Chen et al. (2014) [50], Chen et al. (2015) [51], Clark et al. (2016) [52], Farrokhi et al. (2011) [48], Firanescu et al. (2018) [32], Kallmes et al. (2009) [22], Klazen et al. (2010) [38], Voormolen et al. (2007) [36], Yang et al. (2016) [34].

The results of the analysis of the effectiveness of PV compared to the control group in terms of functionality showed no statistically significant differences in the short (SMD: –0.96; 95%CI: −2.07–0.15), medium (SMD: −1.15; 95%CI: −2.40–0.10), or long term (SMD: −1.40; 95%CI: −3.41–0.62), although significant trends were found for all three time intervals. Figure 4 shows the forest plot of the corresponding analysis.

### 3.5. Efficacy of Percutaneous Vertebroplasty in Quality of Life Improvement

**Figure 5:**
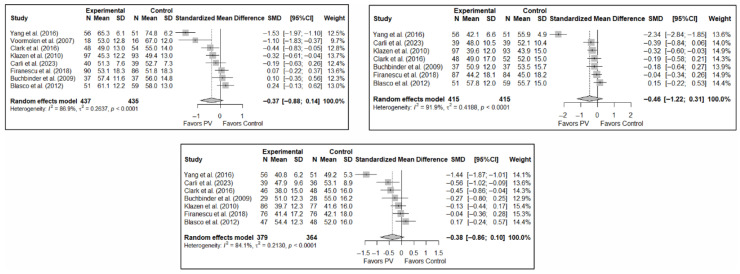
Forest plot of the analysis showing the efficacy of percutaneous vertebroplasty (PV) compared to the control group in terms of quality of life in the short (**top left**), medium (**top right**), and long (**bottom**) term. Overall *p*-values for short-, medium- and long-term analyses were 0.133, 0.194, and 0.103, respectively. Blasco et al. (2012) [49], Buchbinder et al. (2009) [21], Carli et al. (2023) [23], Clark et al. (2016) [52], Firanescu et al. (2018) [32], Klazen et al. (2010) [38], Voormolen et al. (2007) [36], Yang et al. (2016) [34].

As in the case of functionality, the results of the analysis of the effectiveness of PV on quality of life compared to the control showed trends toward statistical significance in favor of the former in the short term (SMD: −0.37; 95%CI: −0.88–0.14), medium term (SMD: −0.46; 95%CI: −1.22–0.31), and long term (SMD: −0.38; 95%CI: −0.86–0.10). Figure 5 shows the forest plot of the corresponding analysis.

### 3.6. Subgroup Analyses

#### 3.6.1. Analysis Based on the Type of Control Group

The results of the analysis of the efficacy of PV in pain relief showed that, in the short term, PV showed benefits compared to placebo (SMD: −0.15; 95%CI: −0.40–−0.10) and CT (SMD: −1.31; 95%CI: −2.57–−0.05). In the medium term, PV showed benefits compared to placebo (SMD: −0.22; 95%CI: −0.40–0.04) but not compared to CT, although there was a trend toward significance (SMD: −1.00; 95%CI: −2.08 to 0.08). In the long term, PV showed significant benefits compared to placebo (SMD: −0.26; 95%CI: −0.46–−0.07) and CT (SMD: −0.89; 95%CI: −1.71–−0.06).

In terms of functionality, it was found that, in the short term, PV showed no benefits compared to placebo (SMD: 0.01; 95%CI: −0.34–0.35) and CT (SMD: −2.02; 95%CI: −4.16–0.13). In the medium term, PV showed no benefits over placebo (SMD: −0.20; 95%CI: −0.58 to 0.19) or CT, although there was a trend toward significance in the latter case (SMD: −2.20; 95%CI: −4.87–0.48). In the long term, PV showed no benefits over placebo (SMD: −0.28; 95%CI: −0.88–0.33) or CT (SMD: −2.55; 95%CI: −7.01–1.90).

Finally, regarding quality of life, it was found that, in the short term, PV showed no benefits compared to placebo (SMD: 0.10; 95%CI: −0.51–0.30) or CT (SMD: −0.66; 95%CI: −1.93–0.61). In the medium term, PV showed no benefits over placebo (SMD: –0.16; 95%CI: −0.39–0.06) or CT (SMD: −0.82; 95%CI: −4.10–2.45). In the long term, PV showed no benefits compared to placebo (SMD: −0.30; 95%CI: −0.68–0.09) or CT (SMD: −0.46; 95%CI: −2.58–1.65).

**Figure 6:**
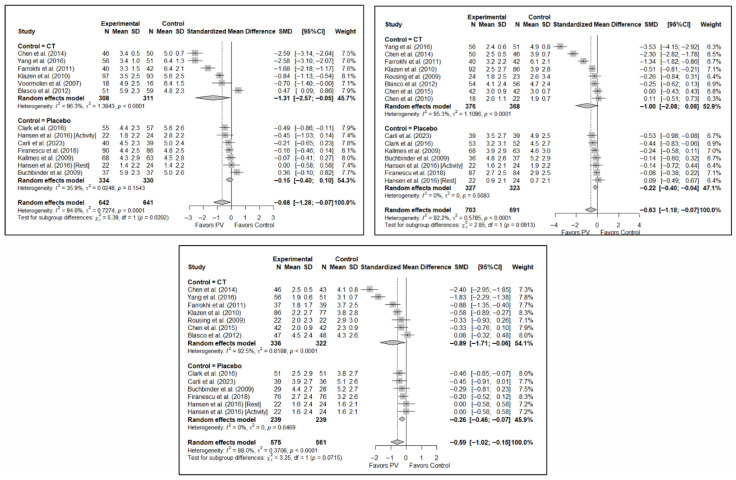
Forest plot showing the subgroup analyses based on the control group for short-term (**top left**), medium-term (**top right**), and long-term (**bottom**) pain. *p*-values for short-, medium- and long-term analyses in the conservative treatment (CT) subgroups were 0.008, 0.027, and 0.008, respectively. *p*-values for short-, medium- and long-term analyses in the placebo subgroups were 0.138, 0.005, and 0.004, respectively. Blasco et al. (2012) [49], Buchbinder et al. (2009) [21], Carli et al. (2023) [23], Chen et al. (2010) [47], Chen et al. (2014) [50], Clark et al. (2016) [52], Farrokhi et al. (2011) [48], Firanescu et al. (2018) [32], Hansen et al. (2016) [31], Kallmes et al. (2009) [22], Klazen et al. (2010) [38], Rousing et al. (2009) [46], Voormolen et al. (2007) [36], Yang et al. (2016) [34].

**Figure A1:**
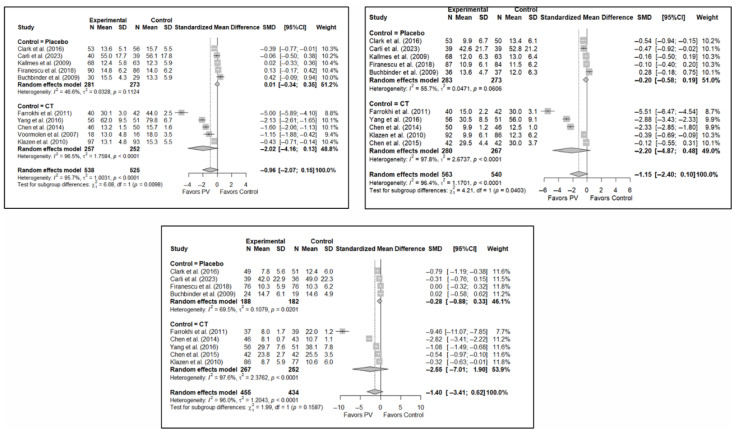
Forest plot showing the subgroup analyses based on the control group for short-term (**top left**), medium-term (**top right**), and long-term (**bottom**) functionality. *p*-values for short-, medium- and long-term analyses in the conservative treatment (CT) subgroups were 0.008, 0.021, and 0.096, respectively. *p*-values for short-, medium- and long-term analyses in the placebo subgroups were 0.964, 0.136, and 0.152, respectively. Buchbinder et al. (2009) [21], Carli et al. (2023) [23], Chen et al. (2014) [50], Chen et al. (2015) [51], Clark et al. (2016) [52], Farrokhi et al. (2011) [48], Firanescu et al. (2018) [32], Kallmes et al. (2009) [22], Klazen et al. (2010) [38], Voormolen et al. (2007) [36], Yang et al. (2016) [34].

**Figure A2:**
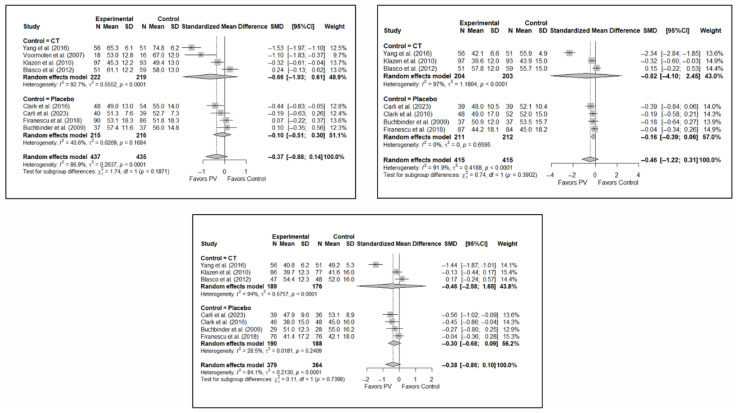
Forest plot showing the subgroup analyses based on the control group for short-term (**top left**), medium-term (**top right**), and long-term (**bottom**) quality of life. *p*-values for short-, medium- and long-term analyses in the conservative treatment (CT) subgroups were 0.008, 0.027, and 0.008, respectively. *p*-values for short-, medium- and long-term analyses in the placebo subgroups were 0.138, 0.005, and 0.004, respectively. Blasco et al. (2012) [49], Buchbinder et al. (2009) [21], Carli et al. (2023) [23], Clark et al. (2016) [52], Firanescu et al. (2018) [32], Klazen et al. (2010) [38], Voormolen et al. (2007) [36], Yang et al. (2016) [34].

Figure 6 shows the forest plot of the subgroup analysis for pain. Figure A1 and Figure A2 in Appendix B show the forest plots for functionality and quality of life, respectively.

#### 3.6.2. Analysis Based on the Assessment of Risk of Bias in Each Study

No significant differences were found in short-term pain depending on whether the risk of bias classification was high or low (SMD: −1.24; 95%CI: −2.89–0.41 and SMD: −0.33; 95%CI: −0.82–0.17, respectively). Nor were there any differences in the medium term, although there was a trend toward significance in both groups, especially for low-risk studies (SMD: −0.95; 95%CI: −2.23–0.33 and SMD: −0.35; 95%CI: −0.71–0.01). In the long term, benefits in favor of PV were found in the low-risk study group (SMD: −0.34; 95%CI: −0.61–−0.08) and there were no statistically significant differences in the high-risk study group (SMD: −0.89; 95%CI: −1.92–0.13).

In terms of functionality and quality of life, benefits in favor of PV were found in the short term in the high-risk group (SMD: −1.32; 95%CI: −2.49–−0.15), but there were no significant differences in the low/uncertain-risk group (SMD: −0.72; 95%CI: −2.79–1.35). In the medium and long term, no significant differences were found in either subgroup. Overall, no statistically significant differences between groups were observed. Similarly, no statistically significant between-group differences were found in quality of life in the short, medium, or long term.

**Figure 7:**
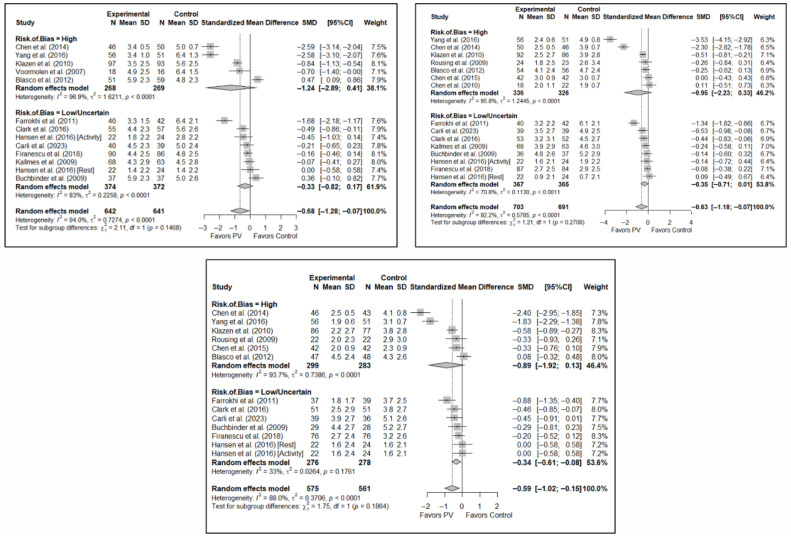
Forest plot showing the subgroup analyses based on the risk of bias for short-term (**top left**), medium-term (**top right**), and long-term (**bottom**) pain. *p*-values for short-, medium- and long-term analyses in the high risk of bias subgroups were 0.037, 0.067, and 0.025, respectively. *p*-values for short-, medium- and long-term analyses in the low/uncertain risk of bias subgroups were 0.113, 0.019, and 0.001, respectively. Blasco et al. (2012) [49], Buchbinder et al. (2009) [21], Carli et al. (2023) [23], Chen et al. (2010) [47], Chen et al. (2014) [50], Clark et al. (2016) [52], Farrokhi et al. (2011) [48], Firanescu et al. (2018) [32], Hansen et al. (2016) [31], Kallmes et al. (2009) [22], Klazen et al. (2010) [38], Rousing et al. (2009) [46], Voormolen et al. (2007) [36], Yang et al. (2016) [34].

**Figure A3:**
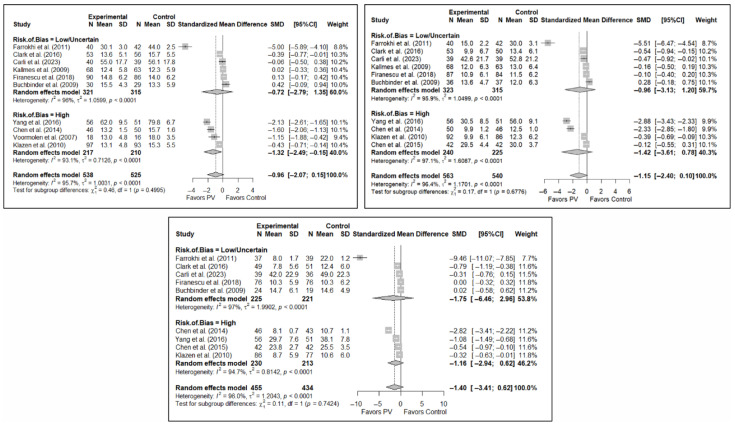
Forest plot showing the subgroup analyses based on the risk of bias for short-term (**top left**), medium-term (**top right**), and long-term (**bottom**) functionality. *p*-values for short-, medium- and long-term analyses in the high risk of bias subgroups were 0.001, 0.039, and 0.035, respectively. *p*-values for short-, medium- and long-term analyses in the low/uncertain risk of bias subgroups were 0.338, 0.226, and 0.255, respectively. Buchbinder et al. (2009) [21], Carli et al. (2023) [23], Chen et al. (2014) [50], Chen et al. (2015) [51], Clark et al. (2016) [52], Farrokhi et al. (2011) [48], Firanescu et al. (2018) [32], Kallmes et al. (2009) [22], Klazen et al. (2010) [38], Voormolen et al. (2007) [36], Yang et al. (2016) [34].

**Figure A4:**
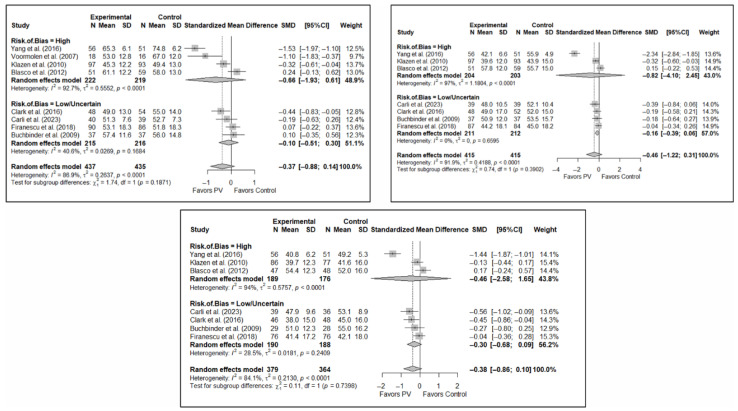
Forest plot showing the subgroup analyses based on the risk of bias for short-term (**top left**), medium-term (**top right**), and long-term (**bottom**) quality of life. *p*-values for short-, medium- and long-term analyses in the high risk of bias subgroups were 0.102, 0.277, and 0.343, respectively. *p*-values for short-, medium- and long-term analyses in the low/uncertain risk of bias subgroups were 0.424, 0.091, and 0.022, respectively. Blasco et al. (2012) [49], Buchbinder et al. (2009) [21], Carli et al. (2023) [23], Clark et al. (2016) [52], Firanescu et al. (2018) [32], Klazen et al. (2010) [38], Voormolen et al. (2007) [36], Yang et al. (2016) [34].

Figure 7 shows the forest plot of the subgroup analysis for pain. Figure A3 and Figure A4 (Appendix B) show the forest plots for functionality and quality of life, respectively.

#### 3.6.3. Analysis Based on the Number of Institutions Involved (Single- vs. Multi-Center)

No statistically significant differences were found depending on whether the study was single- or multicenter in terms of short-term pain (SMD: −1.00; 95%CI: −2.17–0.17 and SMD: −0.31; 95%CI: −0.77–0.15, respectively). In the medium term, significant differences were found in favor of PV in multicenter studies (SMD: −0.29; 95%CI: −0.53–−0.05) and a trend toward significance in single-center studies (SMD: −0.81; 95%CI: −1.68–0.06). However, the test for the difference between subgroups was not significant (*p* = 0.184). In the long term, no statistically significant differences were found between subgroups (*p* = 0.346), with benefits in favor of PV found in both (SMD: –0.68; 95%CI: −1.35–−0.01 and SMD: −0.39; 95%CI: −0.69–−0.10 in the single- and multicenter study groups, respectively). In the three time intervals, a significant decrease in heterogeneity was found for multicenter designs, being especially significant in the medium term (I^2^ = 20.1%) and long term (I^2^ = 1.2%).

In the case of functionality, tests for differences between subgroups showed a trend toward significance in the short term (*p* = 0.058) and statistically significant differences in the medium term (*p* = 0.037), but non-significant in the long term (*p* = 0.157). Despite this, none of the subgroup analyses showed significant differences between the PV and the control group. Heterogeneity was significantly lower in the three time intervals in the case of the subgroup of multi-center studies, most pronounced in the medium term (I^2^ = 56.3%).

In terms of quality of life, the tests for differences between subgroups showed no statistically significant differences in the short, medium, or long terms, nor any benefits in favor of or against PV. Moderate heterogeneity was evident in the multicenter design in the short term (I^2^ = 69.1%) and none in the medium and long term, whilst high heterogeneity was observed in single-center studies across all time intervals.

**Figure 8:**
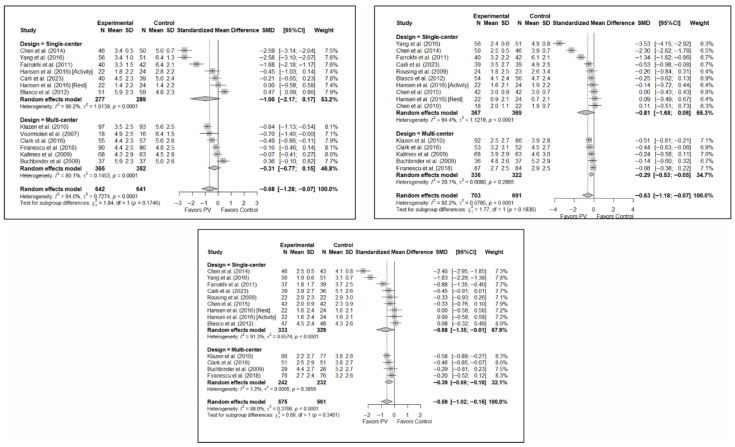
Forest plot showing the subgroup analyses based on the number of institutions involved in each trial for short-term (**top left**), medium-term (**top right**), and long-term (**bottom**) pain. *p*-values for short-, medium- and long-term analyses in single-center study subgroups were 0.037, 0.033, and 0.019, respectively. *p*-values for short-, medium- and long-term analyses in multi-center study subgroups were 0.086, 0.002, and <0.001, respectively. Blasco et al. (2012) [49], Buchbinder et al. (2009) [21], Carli et al. (2023) [23], Chen et al. (2010) [47], Chen et al. (2014) [50], Clark et al. (2016) [52], Farrokhi et al. (2011) [48], Firanescu et al. (2018) [32], Hansen et al. (2016) [31], Kallmes et al. (2009) [22], Klazen et al. (2010) [38], Rousing et al. (2009) [46], Voormolen et al. (2007) [36], Yang et al. (2016) [34].

**Figure A5:**
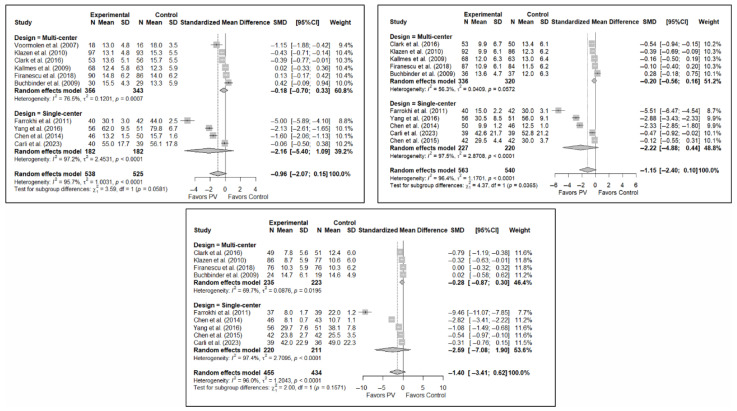
Forest plot showing the subgroup analyses based on the number of institutions involved in each trial for short-term (**top left**), medium-term (**top right**), and long-term (**bottom**) functionality. *p*-values for short-, medium- and long-term analyses in single-center study subgroups were 0.033, 0.019, and 0.096, respectively. *p*-values for short-, medium- and long-term analyses in multi-center study subgroups were 0.307, 0.104, and 0.123, respectively. Buchbinder et al. (2009) [21], Carli et al. (2023) [23], Chen et al. (2014) [50], Chen et al. (2015) [51], Clark et al. (2016) [52], Farrokhi et al. (2011) [48], Firanescu et al. (2018) [32], Kallmes et al. (2009) [22], Klazen et al. (2010) [38], Voormolen et al. (2007) [36], Yang et al. (2016) [34].

**Figure A6:**
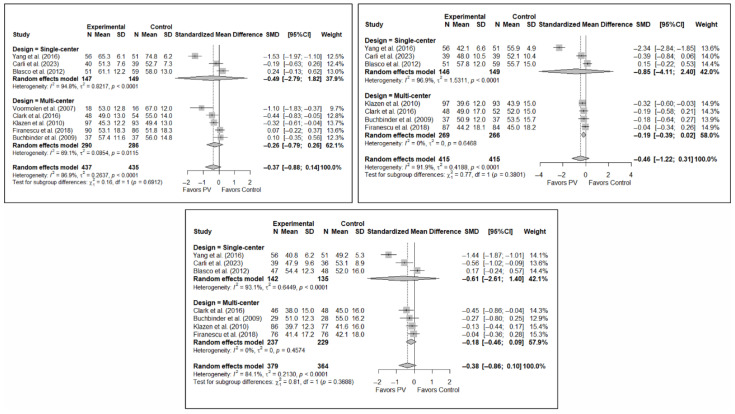
Forest plot showing the subgroup analyses based on the number of institutions involved in each trial for short-term **(top left**), medium-term (**top right**), and long-term (**bottom**) quality of life. *p*-values for short-, medium- and long-term analyses in single-center study subgroups were 0.361, 0.259, and 0.193, respectively. *p*-values for short-, medium- and long-term analyses in multi-center study subgroups were 0.119, 0.032, and 0.005, respectively. Blasco et al. (2012) [49], Buchbinder et al. (2009) [21], Carli et al. (2023) [23], Clark et al. (2016) [52], Firanescu et al. (2018) [32], Klazen et al. (2010) [38], Voormolen et al. (2007) [36], Yang et al. (2016) [34].

Figure 8 shows the forest plot of the corresponding subgroup analysis. Figure A5 and Figure A6 in Appendix B show the forest plot for functionality and quality of life, respectively.

#### 3.6.4. Analysis Based on Geographic Region

With regard to pain, significant differences were found depending on the geographical region in which the short-term study was conducted, with benefits in favor of PV in studies conducted in Eastern countries (SMD: −2.59; 95%CI: −2.63–−2.54) but not in Western countries (SMD: −0.33; 95%CI: −0.73–0.07), although a trend toward significance was observed in the latter subgroup. In the medium term, significant differences were found in Western regions (SMD: −0.35; 95%CI: −0.60–−0.11) but not in Eastern countries (SMD: −1.43; 95%CI: −4.27–1.42), and the test for differences between subgroups was not significant (*p* = 0.23). In the long term, significant differences in favor of PV were also found in studies from Western regions (SMD: −0.33; 95%CI: −0.54–−0.12) but not in those from Eastern countries (SMD: −1.51; 95%CI: −4.17–1.14), with a trend toward statistical significance in the analysis of differences between subgroups (*p* = 0.058).

In terms of functionality, no significant differences were found in favor of or against PV in any of the time intervals, and in terms of quality of life, there was only one study in the group of trials conducted in Eastern countries [34] in which benefits in favor of PV were found in the three follow-up intervals, while no significant differences were found in the subgroup of Western countries.

**Figure 9:**
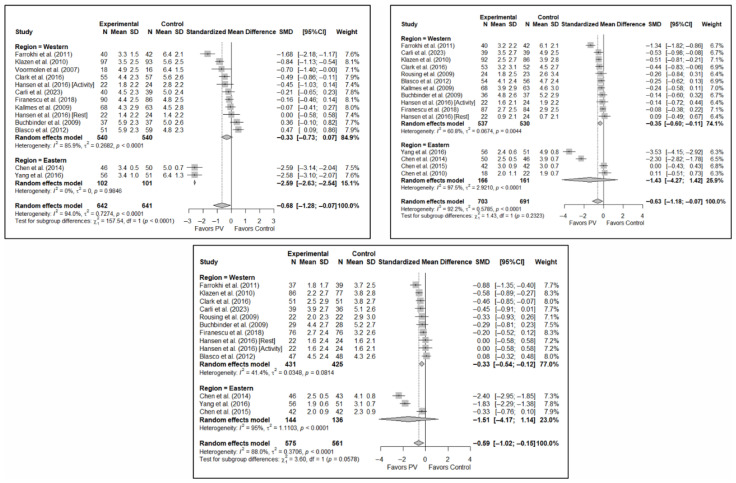
Forest plot showing the subgroup analyses based on the geographic region where the trials were conducted for short-term (**top left**), medium-term (**top right**), and long-term (**bottom**) pain. *p*-values for short-, medium- and long-term analyses in Western subgroups were 0.065, 0.001, and <0.001, respectively. *p*-values for short-, medium- and long-term analyses in Eastern subgroups were <0.001, 0.110, and 0.014, respectively. Blasco et al. (2012) [49], Buchbinder et al. (2009) [21], Carli et al. (2023) [23], Chen et al. (2010) [47], Chen et al. (2014) [50], Clark et al. (2016) [52], Farrokhi et al. (2011) [48], Firanescu et al. (2018) [32], Hansen et al. (2016) [31], Kallmes et al. (2009) [22], Klazen et al. (2010) [38], Rousing et al. (2009) [46], Voormolen et al. (2007) [36], Yang et al. (2016) [34].

**Figure A7:**
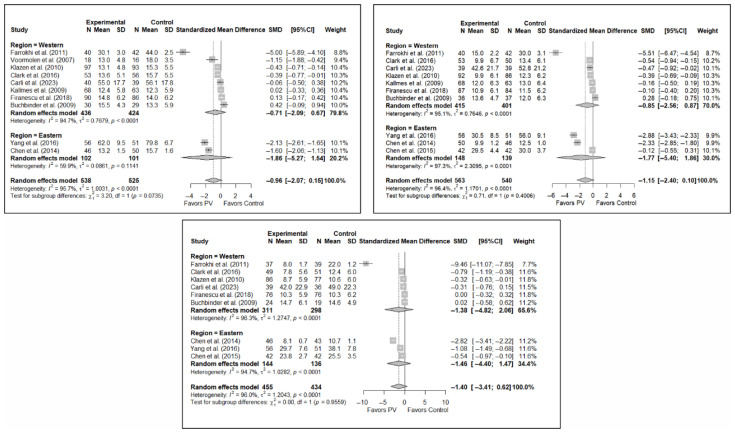
Forest plot showing the subgroup analyses based on the geographic region where the trials were conducted for short-term (**top left**), medium-term (**top right**), and long-term (**bottom**) functionality. *p*-values for short-, medium- and long-term analyses in Western subgroups were 0.195, 0.196, and 0.239, respectively. *p*-values for short-, medium- and long-term analyses in Eastern subgroups were <0.001, 0.036, and 0.031, respectively. Buchbinder et al. (2009) [21], Carli et al. (2023) [23], Chen et al. (2014) [50], Chen et al. (2015) [51], Clark et al. (2016) [52], Farrokhi et al. (2011) [48], Firanescu et al. (2018) [32], Kallmes et al. (2009) [22], Klazen et al. (2010) [38], Voormolen et al. (2007) [36], Yang et al. (2016) [34].

**Figure A8:**
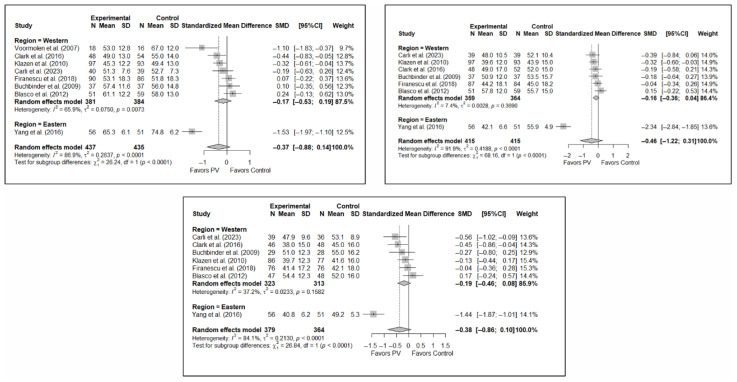
Forest plot showing the subgroup analyses based on the geographic region where the trials were conducted for short-term (**top left**), medium-term (**top right**), and long-term (**bottom**) quality of life. *p*-values for short-, medium- and long-term analyses in Western subgroups were 0.193, 0.053, and 0.067, respectively. *p*-values for short-, medium- and long-term analyses in Eastern subgroups were <0.001 in all cases. Blasco et al. (2012) [49], Buchbinder et al. (2009) [21], Carli et al. (2023) [23], Clark et al. (2016) [52], Firanescu et al. (2018) [32], Klazen et al. (2010) [38], Voormolen et al. (2007) [36], Yang et al. (2016) [34].

Figure 9 shows the forest plot of the corresponding subgroup analysis. Figure A7 and Figure A8 in Appendix B show the forest plot for functionality and quality of life, respectively.

#### 3.6.5. Analysis Based on Fracture Chronicity

Significant differences were found depending on the chronicity of the fracture, in the short term, if the chronicity was less than 8 weeks (SMD: −0.41; 95%CI: −0.82–0.00) but not if it was greater than 8 weeks (SMD: −0.87; 95%CI: −1.91–0.18). In the medium term, significant differences were found in OVFs <8 weeks (SMD: −0.27; 95%CI: −0.51–−0.02), but not in those lasting >8 weeks (SMD: −0.90; 95%CI: −1.85–0.05). In the long term, significant differences were found in both groups, i.e., <8 weeks (SMD: –0.32; 95%CI: −0.57–−0.08) vs. >8 weeks (SMD: −0.86; 95%CI: −1.70–−0.03), but heterogeneity was high in the former and low in the latter.

In terms of functionality, no statistically significant differences were found in favor of or against PV in any of the follow-up intervals, and the tests for differences between subgroups were not statistically significant. Similar findings were also observed in the case of quality of life.

**Figure 10:**
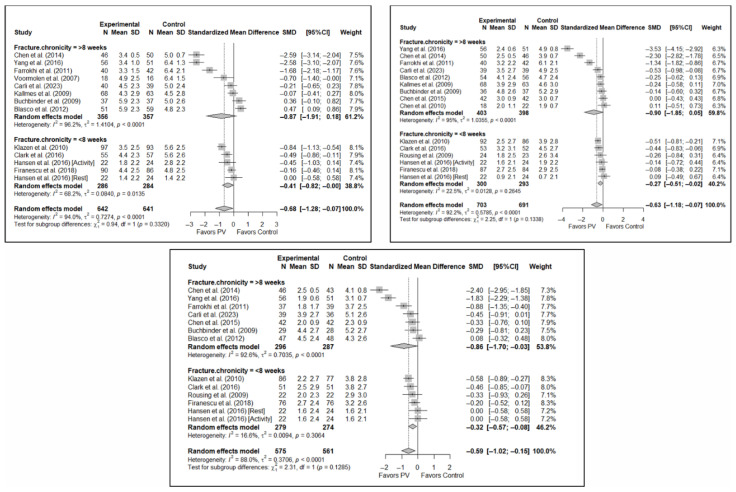
Forest plot showing the subgroup analyses based on fracture chronicity for short-term (**top left**), medium-term (**top right**), and long-term (**bottom**) pain. *p*-values for short-, medium- and long-term analyses in >8 weeks subgroups were 0.051, 0.028, and 0.011, respectively. *p*-values for short-, medium- and long-term analyses in <8 weeks subgroups were 0.007, 0.011, and 0.002, respectively. Blasco et al. (2012) [49], Buchbinder et al. (2009) [21], Carli et al. (2023) [23], Chen et al. (2010) [47], Chen et al. (2014) [50], Clark et al. (2016) [52], Farrokhi et al. (2011) [48], Firanescu et al. (2018) [32], Hansen et al. (2016) [31], Kallmes et al. (2009) [22], Klazen et al. (2010) [38], Rousing et al. (2009) [46], Voormolen et al. (2007) [36], Yang et al. (2016) [34].

**Figure A9:**
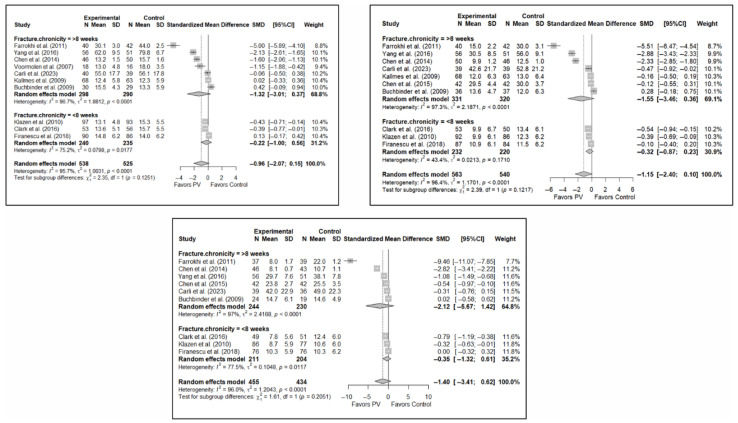
Forest plot showing the subgroup analyses based on fracture chronicity for short-term (**top left**), medium-term (**top right**), and long-term (**bottom**) functionality. *p*-values for short-, medium- and long-term analyses in >8 weeks subgroups were 0.053, 0.044, and 0.108, respectively. *p*-values for short-, medium- and long-term analyses in <8 weeks subgroups were 0.225, 0.011, and 0.112, respectively. Buchbinder et al. (2009) [21], Carli et al. (2023) [23], Chen et al. (2014) [50], Chen et al. (2015) [51], Clark et al. (2016) [52], Farrokhi et al. (2011) [48], Firanescu et al. (2018) [32], Kallmes et al. (2009) [22], Klazen et al. (2010) [38], Voormolen et al. (2007) [36], Yang et al. (2016) [34].

**Figure A10:**
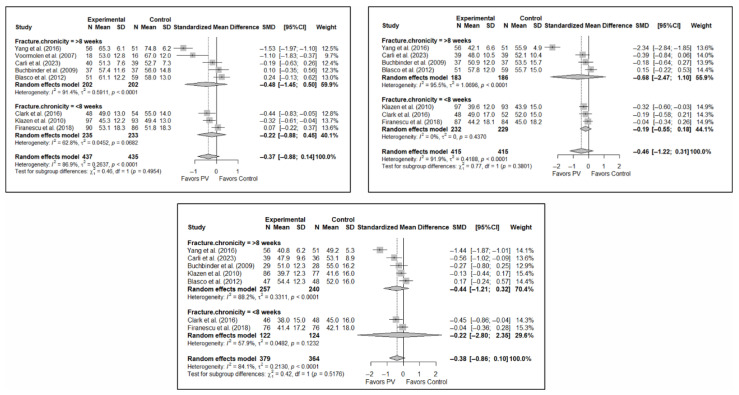
Forest plot showing the subgroup analyses based on fracture chronicity for short-term (**top left**), medium-term (**top right**), and long-term (**bottom**) quality of life. *p*-values for short-, medium- and long-term analyses in >8 weeks subgroups were 0.170, 0.221, and 0.108, respectively. *p*-values for short-, medium- and long-term analyses in <8 weeks subgroups were 0.162, 0.046, and 0.273, respectively. Blasco et al. (2012) [49], Buchbinder et al. (2009) [21], Carli et al. (2023) [23], Clark et al. (2016) [52], Firanescu et al. (2018) [32], Klazen et al. (2010) [38], Voormolen et al. (2007) [36], Yang et al. (2016) [34].

Figure 10 shows the forest plot of the corresponding subgroup analysis. Figure A9 and Figure A10 in Appendix B show the forest plots for functionality and quality of life, respectively.

### 3.7. Sensitivity and Publication Bias Analysis

The sensitivity analysis performed on the overall pain estimator found that the elimination of the study by Yang et al. (2016) [34] had a slightly greater influence in the short and medium terms than the other studies (decrease in I^2^ from 94% to 91.8% in the short term and from 92.2% to 84.4% in the medium term), while in the long term, the exclusion of the study by Chen et al. (2014) [50] resulted in a significant reduction in heterogeneity (from 88% to 79.6%). In the case of functionality, no study had a predominant influence, with the study by Farrokhi et al. (2011) [48] producing the greatest decrease in I^2^, especially in the long term (decrease in I^2^ from 96.0% to 91.5%). Finally, in terms of quality of life, the exclusion of the study by Yang et al. (2016) [34] in the medium and long term led to a clear decrease in heterogeneity (from 91.9% to 7.4% in the medium term and from 84.1% to 37.2% in the long term).

**Figure 11:**
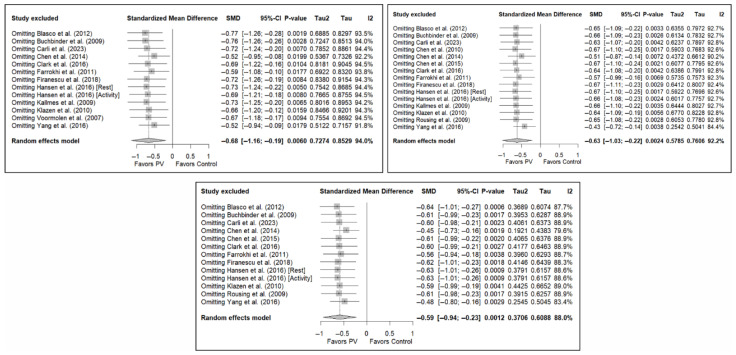
Sensitivity analyses of studies evaluating pain in the short (**top left**), medium (**top right**), and long (**bottom**) terms. Blasco et al. (2012) [49], Buchbinder et al. (2009) [21], Carli et al. (2023) [23], Chen et al. (2010) [47], Chen et al. (2014) [50], Clark et al. (2016) [52], Farrokhi et al. (2011) [48], Firanescu et al. (2018) [32], Hansen et al. (2016) [31], Kallmes et al. (2009) [22], Klazen et al. (2010) [38], Rousing et al. (2009) [46], Voormolen et al. (2007) [36], Yang et al. (2016) [34].

**Figure A11:**
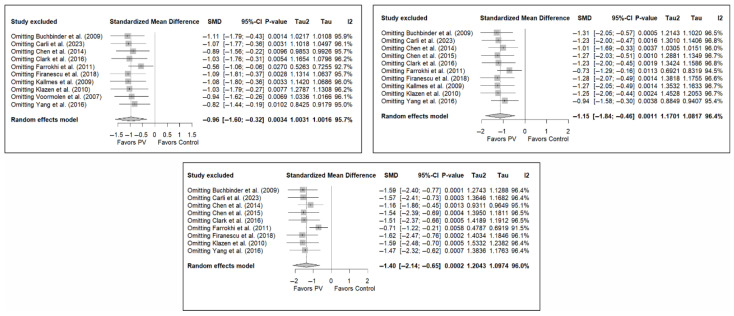
Sensitivity analysis for the variable functionality in the short, medium, and long term. Buchbinder et al. (2009) [21], Carli et al. (2023) [23], Chen et al. (2014) [50], Chen et al. (2015) [51], Clark et al. (2016) [52], Farrokhi et al. (2011) [48], Firanescu et al. (2018) [32], Kallmes et al. (2009) [22], Klazen et al. (2010) [38], Voormolen et al. (2007) [36], Yang et al. (2016) [34].

**Figure A12:**
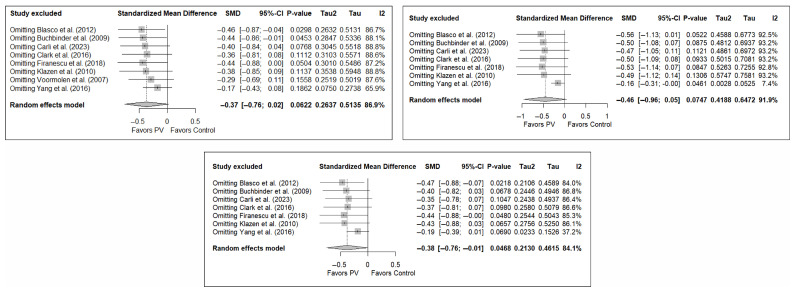
Sensitivity analysis for the variable quality of life in the short, medium, and long term. Blasco et al. (2012) [49], Buchbinder et al. (2009) [21], Carli et al. (2023) [23], Clark et al. (2016) [52], Firanescu et al. (2018) [32], Klazen et al. (2010) [38], Voormolen et al. (2007) [36], Yang et al. (2016) [34].

Figure 11 shows the results of the sensitivity analyses for pain, and Figure A11 and Figure A12 in Appendix B show the sensitivity analyses for functionality and quality of life, respectively.

**Figure 12:**
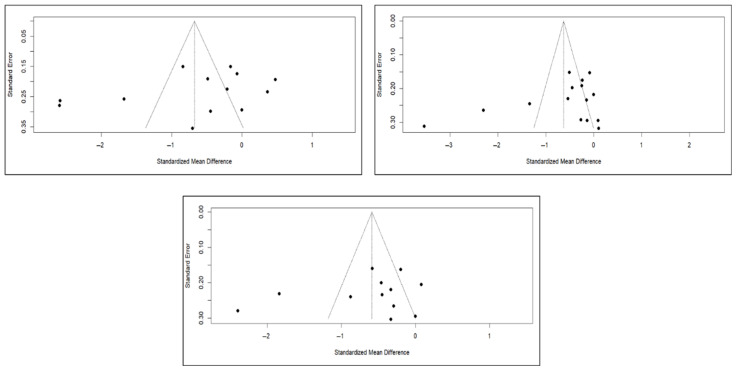
Funnel diagrams of studies based on the variable of short-term (**left**), medium-term (**right**), and long-term (**bottom**) pain.

**Figure A13:**
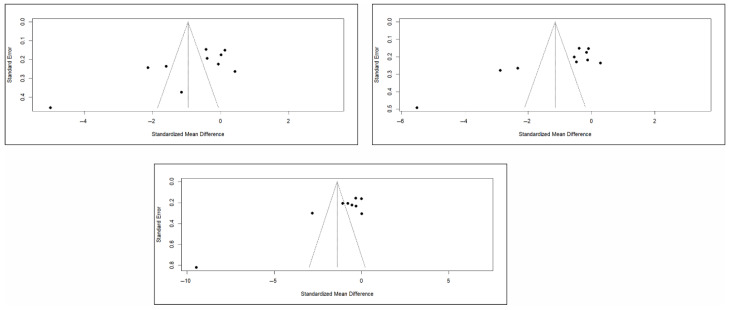
Funnel plots of studies based on functionality in the short, medium, and long term.

**Figure A14:**
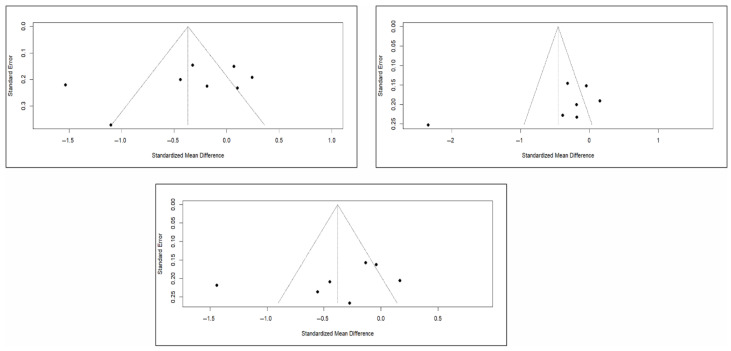
Funnel plots of studies based on quality of life in the short, medium, and long term.

Finally, the analysis of publication bias using funnel plots showed an erratic distribution of studies, with no clear signs of publication bias in pain and quality of life. However, in the case of functionality, an inverted pyramid distribution was evident, suggesting possible publication bias. Figure 12 shows the corresponding funnel diagram for the pain variable, and Figure A13 and Figure A14 (Appendix C) show the diagrams corresponding to functionality and quality of life, respectively.

The results of the Egger test for the main variables at different follow-up intervals showed no evidence of publication bias in the comparisons of pain or quality of life. However, significant asymmetry was detected in functionality in the three time intervals, suggesting the existence of publication bias, although the number of studies included in some comparisons was limited (<10), which may affect the stability of the test (Appendix C).

## 4. Discussion

This systematic review with a meta-analysis sought to update existing knowledge on the efficacy of PV by means of a quantitative synthesis of RCTs compared with placebo and CT. Although there are previous similar studies that have addressed this issue, this meta-analysis is particularly relevant for several reasons. Firstly, we pooled available results in terms of pain, functionality, and quality of life, including the recent VERTOS V clinical trial [23], which is particularly important given the scarcity of clinical trials published since 2018 [32]. Secondly, we included trials found in sources other than the most common biomedical databases (in particular, the Chinese trials by Chen et al., 2010 [47] and by Chen et al., 2015 [51]), which have been previously analyzed only in a few previous meta-analyses [24]. Third, we obtained pooled estimates for comparisons encompassing both placebo and CT groups, as well as following stratification by different potentially relevant variables in subgroup analyses. Fourthly, we conducted separated analyses of data from one individual study (Hansen et al., 2016 [31]) to account for outcomes measured at rest and during forward bending. Fifth, the threshold chosen for differentiating between acute and chronic OVFs (8 weeks) was slightly higher than that described in previous studies [24,25,52] (6 weeks). In addition, we performed different subgroup and sensitivity analyses that showed potential sources of heterogeneity and confounding factors that need to be addressed in future studies. Finally, we followed some methodological approaches that simplify the interpretation of results and reduce the bias derived from missing data through a rigorous application of estimation techniques described in the scientific literature.

The results obtained reinforce the usefulness of PV in the treatment of OVFs, particularly due to the benefits in terms of pain relief, demonstrated in the short, medium, and long term in overall analyses. In subgroup analyses based on the control group, such an advantage was more pronounced in studies compared PV with CT in the short term (SMD, −1.31; 95%CI, −2.57–−0.05), suggesting that part of the benefit may derive from procedural or contextual effects that go beyond PV itself. However, the magnitude of improvement in the placebo-controlled trials still indicates a specific therapeutic contribution of PV. Notably, clear trends toward significance were observed in the medium and short term in studies using CT and placebo as control groups, respectively. These findings are consistent with previous studies. For example, a previous meta-analysis [24] found benefits in favor of PV compared to the control group in all time intervals, as well as in the subgroup analyses, with the exception of short and medium term pain in studies compared with placebo (trend toward significance in medium term pain).

Regarding functionality, no statistically significant differences were found, although trends toward significance favoring PV were observed in all follow-up intervals, overall and when stratifying based on the nature of the control group. Previous studies observed statistically significant differences favoring PV in some follow-up intervals. For instance, Lainez Ramos-Bossini et al. (2021) [24], Zuo et al. [41], Zhu et al. (2019) [53], and Lou et al. (2019) [26] found differences in favor of PV at all three time intervals in studies compared with CT. However, it should be noted that these meta-analyses found significant variability in results for some time intervals and that other meta-analyses, such as that by Sanli et al. (2020) [54], found no significant differences compared to placebo (although they did find differences compared to CT). Moreover, the absence of statistically significant differences in functionality may be explained by the limited number of available studies and by the heterogeneity of measurement instruments.

Concerning quality of life, no significant overall differences were found, but trends toward significance in favor of PV were observed in studies compared with placebo. Notably, a previous meta-analysis [24] found differences in favor of PV in the short term in the subgroup of trials compared with short-term CT, as well as in the overall results when the three follow-up intervals were combined in both subgroups (placebo and CT). In addition, a recent meta-analysis [55] that included only the conservative approach as a comparative arm found statistically significant differences in favor of PV in short-term (1–2 weeks) and medium-term (3 months) quality of life, although there are some methodological concerns, such as the joint inclusion of the RMDQ and QUALEFFO scales, which aim to evaluate different psychological constructs.

The differences between the findings of this meta-analysis and previous ones can be explained by several reasons beyond having included the recent VERTOS V trial and separated the VOPE trial. For instance, in most previous meta-analyses (e.g., [24,27]) both mean and SD values for each group and values of the difference in means between groups (and their respective SDs) with respect to baseline were included as effect measures of individual studies, a legitimate methodological decision [35] that allows the integration of data on baseline changes in the quantitative synthesis. In the present study, we opted to systematically use the mean and SD values for the analysis of individual effects for each study to ensure uniform analysis across the included trials (some of which did not report baseline changes) and to facilitate interpretability of forest plots, especially for non-expert readers. Notably, such decision is methodologically appropriate and was grounded on the assumption that no significant baseline differences between groups were present in the trials, which was verified during data extraction. This may justify slight variations in the value of the pooled results of the estimators compared to previous meta-analyses, although the overall trends are clear, particularly regarding pain relief.

The observed dissociation between pain relief and the absence of significant improvement in functionality and quality of life deserves further consideration. This difference is likely explained, at least in part, by the design of the included RCTs because in all of them pain was the primary outcome, while functionality and quality of life were typically secondary outcomes, and therefore these studies were not specifically powered to detect changes in the latter domains. In addition, all outcomes were analyzed through SMDs as different scales were used across trials but, for pain, the only variation was between scales considering wider ranges (e.g., 0–100 [31] instead of the usual 0–10), and one case using a VAS range of 1–10 [48]; conversely, functionality and quality of life scales included a wider variability (e.g., RMDQ vs. ODI or different versions of QUALEFFO), which may have increased variability and attenuated statistical significance. However, beyond methodological nuances, several clinical explanations can be proposed; pain reduction following PV may not restore functional capacity and quality of life, at least in the short-term, as mobility and daily living performance depend on many other psychological and physical factors (e.g., spinal deformities, muscle deconditioning, fear of movement), as well as sociodemographic variables [56]. Moreover, improvements in pain often precede measurable gains in activity or self-perceived well-being, especially in elderly or frail populations. Therefore, functionality and quality of life reflect more complex constructs than pain intensity alone, and their recovery may require complementary interventions such as physiotherapy or multidisciplinary rehabilitation programs [57].

The case of fracture chronicity is particularly noteworthy. Previous studies have found that PV offers better results in more recent OVFs. Although there is no explicit consensus on the time threshold at which a fracture is considered recent, most previous studies consider it to be in the range of 6 to 8 weeks [41]. From a meta-analytical point of view, this difference of just two weeks can lead to changes when performing comparative analyses, since some studies change from one time interval to another (i.e., short to medium term) based on inclusion criteria (see Table 1). Of note, previous meta-analyses have used both 6-week [24,25,26] and 8-week [41] cutoffs. Our a priori decision on an 8-week threshold aimed at providing complementary information by using a timepoint less explored in the previous literature and is supported by recent studies highlighting that central pain sensitization in OVFs occurs after 8 weeks from fracture onset [42].

The results obtained are of great interest, as we found that, for studies that included acute OVFs (i.e., <8 weeks), there were significant differences in favor of PV in the short, medium, and long terms, while they were only significant in the long term in chronic OVF. These results contrast with those from a previous meta-analysis using a 6-week threshold, which found significant advantages favoring PV only in the short-term [24]. These findings highlight the importance of the chronicity of OVFs, in line with previous studies, notably the trial by Clark et al. (2016), which only included OVFs <6 weeks [52]. For functionality and quality of life, no significant differences were found in any of the follow-up intervals based on the chronicity of the fracture.

With regard to this last point, it is worth noting the inclusion in this meta-analysis of the study by Carli et al. [23], which is an active-controlled trial focusing on chronic OVFs (>3 months from diagnosis). Although this type of procedure is not a placebo per se (and, in fact, it has been suggested that the way in which the placebo is administered could introduce certain biases [24]), its therapeutic effect is generally regarded as negligible, given its purely local and short-lived action. It is also worth mentioning the interest of subgroup analyses based on the number of institutions involved and in the risk of bias, since in both cases, our results indicate that, in general, better designs show results more favorable to PV, something that should also considered from a qualitative point of view, since the external validity of such studies is more robust. Finally, subgroup analysis based on geographical region, in addition to being limited by the few Eastern studies on some of the variables, showed some inconsistent results that do not allow reliable conclusions to be drawn. However, regarding pain, we found significant results favoring PV in studies conducted in Western countries (i.e., in medium and long terms). These findings deserve further consideration in future studies, as they could be due to variations in selection or follow-up protocols (e.g., rehabilitation in CT strategies), as well as differences in healthcare access that are difficult to ascertain through indirect analyses.

The results of this study should be considered in a broader context that takes into account other outcome variables not addressed in this meta-analysis, including potential procedure-related differences (e.g., cement type or technical procedures), but especially those related to the safety profile and economic benefits of PV. Regarding the former, the main variables to consider are the risk of developing metachronous OVFs and complications associated with the procedure. In the first case, as with efficacy, there are conflicting positions and results in the literature. A large number of observational studies and clinical trials have shown mixed results: from no significant differences between PV and CT [25] to a higher risk in PV [27], and even a higher risk in CT [58]. However, published clinical trials have focused more on efficacy than on safety, reinforcing the need for more data to available evidence. In our opinion, most controversies in this regard may be related to the role of asymptomatic cement leakage, which has been considered by some authors as a complication to account for, while others (including us) do not. In fact, in a previous meta-analysis on the safety of PV focused on serious adverse effects (e.g., those requiring hospitalization) [25], we found that PV is associated with a lower odds ratio of suffering complications. Moreover, in OVFs treated during the first 6 weeks, PV decreases the likelihood of developing metachronous fractures. Regarding the economic benefits of PV, most previous meta-analyses suggest that PV is cost-effective compared to CT [59,60], but there are still several gaps regarding the magnitude of economic benefits. Taken together, our results support a selective, evidence-based indication of PV focused on early, symptomatic fractures refractory to conservative therapy, rather than its systematic application or complete dismissal. This aligns with guidelines such as NICE and CIRSE [61], which highlight the importance of selecting recent fractures with refractory pain not responding to CT.

This study accounts for several limitations, among which it is worth highlighting, first, the low number (6) of placebo-controlled trials and high methodological heterogeneity of the different trials included, as shown by the I^2^ (and τ^2^) values in most comparisons. Second, some of the main explored variables were reported by a limited number of trials, precluding robust analyses at certain follow-up intervals. Third, the criterion for establishing the chronicity cutoff point at 8 weeks (instead of 6 weeks) in the subgroup analysis and the choice of using means in cases where values of change from baseline were available may limit the comparability of our pooled estimates with respect to previous meta-analyses. Similarly, we applied several approaches to handle missing data. Although this might have introduced a slight degree of uncertainty, the direction and consistency of results remained stable in sensitivity analyses. Another finding that should be considered is related to the cues of publication bias observed in functionality; however, in this meta-analysis our findings were not robustly suggestive of benefits favoring PV -despite a trend toward significance was found-, thus a positive publication bias in this outcome would likely make this lack of association more evident. Also, there were less than 10 studies in most publication bias analysis, which imposes a restriction on their reliability. Finally, it should be noted that, although quantitative benefits in pain relief were observed, this does not necessarily justify the routine implementation of PV in clinical practice -despite being one, if not the most relevant variable usually considered in real-world clinical practice-; other potential relevant outcomes, including not only pain, functionality and quality of life but also safety and cost–benefit balance need to be taken into account. Therefore, our results need to be interpreted in a wider context that requires further studies specifically addressing such aspects.

## 5. Conclusions

The available evidence shows benefits of vertebroplasty over placebo and conservative treatment in pain relief of osteoporotic vertebral fractures. However, no clear differences were found in terms of functionality and quality of life improvement. There is a high heterogeneity that justifies the conduct of future, appropriately designed clinical trials to definitively demonstrate the usefulness of this procedure in clinical practice.

## Figures and Tables

**Table 1:** Characteristics of the studies included in the meta-analysis. ^a^ Maximum follow-up (in days unless otherwise specified). ^b^ In months unless otherwise specified. ^c^ Interquartile range values between brackets. PV, percutaneous vertebroplasty. CT, conservative treatment. F-U, follow-up. M, male. F, female. VAS, Visual Analog Scale. ODI, Oswestry Disability Index. RMDQ, Roland-Morris Disability Questionnaire. QUALEFFO, Quality of Life Questionnaire of the European Foundation for Osteoporosis. W, weeks. Note: several trials reported follow-up or complementary data from the same trial in different publications that can be consulted in Supplementary File S2.

Study	Group	*N*	Age	M/F Ratio	Symptoms Duration ^a^	F-U ^b^	Initial VAS	Initial RMDQ	Initial ODI	Initial QUALEFFO
Voormolen et al. (2007) [36]	PV	18	72 (59–84)	4/14	85 (47–138)	2 W	7.1 (5–9)	15.7 (8–22)	-	60 (37–86)
	CT	16	74 (55–88)	2/14	76 (46–141)	2 W	7.6 (5–10)	17.8 (9–24)	-	67 (38–86)
Rousing et al. (2009) [46]	PV	25	80 (65–96)	6/19	8.4 (3.7–13)	12	7.5 (6.6–8.4)	-	-	-
	CT	24	80 (71–93)	3/21	6.7 (2.1–11.4)	12	8.8 (8.2–9.3)	-	-	-
Kallmes et al. (2009) [22]	PV	68	73.4 (9.4)	15/53	16 (10.36) ^c^ W	1	6.9 (2.0)	16.6 (3.8)	-	-
	Placebo	63	74.3 (9.6)	17/46	20 (8–38) ^c^ W	1	7.2 (1.8)	17.5 (4.1)	-	-
Buchbinder et al. (2009) [21]	PV	38	74.2 (14)	7/31	9 (3.8–13) ^c^ W	24	7.4 (2.1)	17.3 (2.8)	-	56.9 (13.4)
	Placebo	40	78.9 (9.5)	9/31	9.5 (3–17) ^c^ W	24	7.1 (2.3)	17.3 (2.9)	-	59.6 (17.1)
Chen et al. (2010) [47]	PV	18	77.5 (0.8)	4/14	<6 W	3	7.8 (1.2)	-	-	-
	CT	22	76.3 (0.5)	6/16	<6 W	3	8.1 (0.8)	-	-	-
Klazen et al. (2010) [38]	PV	101	75.2 (9.8)	31/70	29.3 (17.1)	12	7.8 (1.5)	18.6 (3.6)	-	58.7 (13.5)
	CT	101	75.4 (8.4)	31/70	26.8 (16.0)	12	7.5 (1.6)	17.2 (4.2)	-	54.7 (14.4)
Farrokhi et al. (2011) [48]	PV	40	72 (59–90)	10/30	27 (4–50) ^c^ W	36	8.4 (1.6)	-	51.2 (2.2)	-
	CT	42	74 (55–87)	12/30	30 (6–54) ^c^ W	36	7.2 (1.7)	-	47.1 (2.8)	-
Blasco et al. (2012) [49]	PV	64	71.3 (10)	17/47	140.3 (96.1)	12	7.21 (0.3)	-	-	65.2 (2.2)
	CT	61	75.3 (8.5)	11/50	143.1 (130.3)	12	6.3 (0.4)	-	-	59.2 (2.2)
Chen et al. (2014) [50]	PV	46	64.6 (9.1)	14/32	7.1 (3)	12	6.5 (0.9)	18.6 (1.8)	59.9 (2.2)	-
	CT	47	66.5 (9.1)	13/30	6.8 (2.5)	12	6.4 (0.9)	16.7 (1.3)	57.9 (1.9)	-
Chen et al. (2015) [51]	PV	42	67 (8.4)	18/24	-	34.7	7.3 (1.0)	48.8 (6.8)	-	
	CT	42	66.1 (8.7)	19/23	-	34.7	7.5 (0.8)	48.9 (7.3)	-	
Hansen et al. (2016) [31]	PV	22	70.6 (54–90)	4/18	74.7 (4.6)	12	5.3 (0.4)	-	-	-
	Placebo	24	69.3 (53–84)	2/22	76.1 (4.4)	12	4.6 (0.46)	-	-	-
Yang et al. (2016) [34]	PV	56	77.1 (6.0)	20/36	5.5 (3.9)	12	7.5 (1.1)	-	80.2 (9.9)	78.1 (8.1)
	CT	51	76.2 (5.6)	18/33	5.6 (3.8)	12	7.7 (1.1)	-	81.5 (9.7)	77.5 (8.6)
Clark et al. (2016) [52]	PV	61	80 (7)	13/48	2.8 (1.6) W	6	8.1 (1.8)	19.5 (3.5)	-	65.4 (11.4)
	Placebo	59	81 (7)	19/40	2.4 (1.4) W	6	8.2 (1.5)	19.8 (3.7)	-	67.7 (11.2)
Firanescu et al. (2018) [32]	PV	90	74.7 (10.7)	23/67	43 (29–52)	12	7.7 (1.4)	18.0 (4.5)	-	68.4 (17.1)
	Placebo	86	76.9 (8.1)	20/66	36 (24–51)	12	7.9 (1.6)	17.8 (4.7)	-	69.7 (17.9)
Carli et al. (2023) [23]	PV	40	69 (10)	13/27	176 (43–907)	12	7.6 (1.8)	64.7 (20)	-	61.8 (9.3)
	Placebo	40	71 (10)	13/27	185 (68–1165)	12	7.3 (1.5)	63.4 (17.9)	-	60.9 (8.8)

## Data Availability

The original contributions presented in the study are included in the article/Supplementary Material, further inquiries can be directed to the corresponding author.

## Supplementary Materials

The following supporting information can be downloaded at: https://www.mdpi.com/article/10.3390/diagnostics15212684/s1, Supplementary File S1. PRISMA checklist. Supplementary File S2. Studies excluded from the meta-analysis following full-text reading. Supplementary File S3. Summary assessment of risk of bias according to the Cochrane Risk of Bias Tool v. 2. Supplementary File S4. Details on domain-specific judgments of the Cochrane Risk of Bias Tool v.2.

## Appendix A. Search Strategies

### Appendix A.1. Search Strategy in the PubMed Database

(((“vertebroplasty”[MeSH Terms] OR “cementoplasty”[Text Word] OR “vertebral augmentation”[Text Word]) AND “Osteoporosis”[MeSH Terms] AND “Spinal Fractures”[MeSH Terms]) OR ((“vertebroplasty”[MeSH Terms] OR “cementoplasty”[Text Word] OR “vertebral augmentation”[Text Word]) AND “Spinal Fractures”[MeSH Terms] AND (“conservative treatment”[Text Word] OR “conservative management”[Text Word] OR “usual care”[Text Word] OR “non surgical treatment”[Text Word] OR “non surgical management”[Text Word] OR “standard medical therapy”[Text Word] OR “placebo”[Text Word] OR “sham”[Text Word]))) AND 1900/01/01:2025/08/11[Date-Publication].

### Appendix A.2. Search Strategy in the EMBASE Database

(‘percutaneous vertebroplasty’/exp OR ‘percutaneous vertebroplasty’ OR ‘cementoplasty’/exp OR ‘vertebral augmentation’/exp) AND osteoporo* AND (‘conservative treatment’/exp OR ‘placebo’/exp) AND [1-1-1950]/sd NOT [11-8-2025]/sd.

### Appendix A.3. Search Strategy in the Web of Science Database

TS = (vertebroplasty OR cementoplasty OR “vertebral augmentation”) AND TS = (osteopor*) AND TS = (“conservative treatment” OR “conservative management” OR “usual care” OR “non-surgical treatment” OR “non-surgical management” OR “standard medical therapy” OR placebo OR sham) AND PY = (2000–2025) (search filtered by publication date from Between 1 January 1900 and 11 August 2025).

### Appendix A.4. Search Strategy in the CENTRAL Database

((vertebroplasty OR cementoplasty OR “vertebral augmentation”) AND (osteoporosis*) AND (“conservative treatment” OR “conservative management” OR “usual care” OR “non surgical treatment” OR “non surgical management” OR “standard medical therapy” OR placebo OR sham)):ti,ab,kw (Cochrane Library publication date Between 1 January 1900 and 11 August 2025).

## Appendix B. Subgroup Analyses

All forest plots in Appendix B follow the same criteria as the figures in the body of the manuscript: short term on the left, medium term on the right, and long term below.

## Appendix C. Funnel Plots and Egger’s Test Results

Results of Egger’s tests for the main variables at different follow-up intervals. df, degrees of freedom. SE, standard error.

**Pain (short-term)** Test result: *t* = −1.23, df = 11, *p*-value = 0.2447 Bias estimate: −5.3138 (SE = 4.3239) **Pain (medium-term)** Test result: *t* = −1.37, df = 13, *p*-value = 0.1944 Bias estimate: −5.0604 (SE = 3.6982) **Pain (long-term)** Test result: *t* = −0.64, df = 11, *p*-value = 0.5362 Bias estimate: −2.4923 (SE = 3.9034) **Functionality (short-term)** Test result: *t* = −2.50, df = 8, *p*-value = 0.0367 Bias estimate: −10.0162 (SE = 4.0003) **Functionality (medium-term)** Test result: *t* = −3.67, df = 8, *p*-value = 0.0063 Bias estimate: −14.2904 (SE = 3.8903) **Functionality (long-term)** Test result: *t* = −3.65, df = 7, *p*-value = 0.0082 Bias estimate: −11.6978 (SE = 3.2039) **Quality of Life (short-term)** Test result: *t* = −1.08, df = 6, *p*-value = 0.3224 Bias estimate: −4.2649 (SE = 3.9560) **Quality of Life (medium-term)** Test result: *t* = −1.36, df = 5, *p*-value = 0.2331 Bias estimate: −8.5357 (SE = 6.2939) **Quality of Life (long-term)** Test result: *t* = −1.05, df = 5, *p*-value = 0.3414 Bias estimate: −5.5917 (SE = 5.3204)
