# Supplementary figures and images for: Efficacy of Percutaneous Vertebroplasty Versus Placebo and Conservative Treatment in Osteoporotic Vertebral Fractures: An Updated Systematic Review and Meta-Analysis of Randomized Clinical Trials
Source: Diagnostics (Basel). 2025 Oct 23;15(21):2684. doi: 10.3390/diagnostics15212684 (PMC12607622; doi:10.3390/diagnostics15212684)

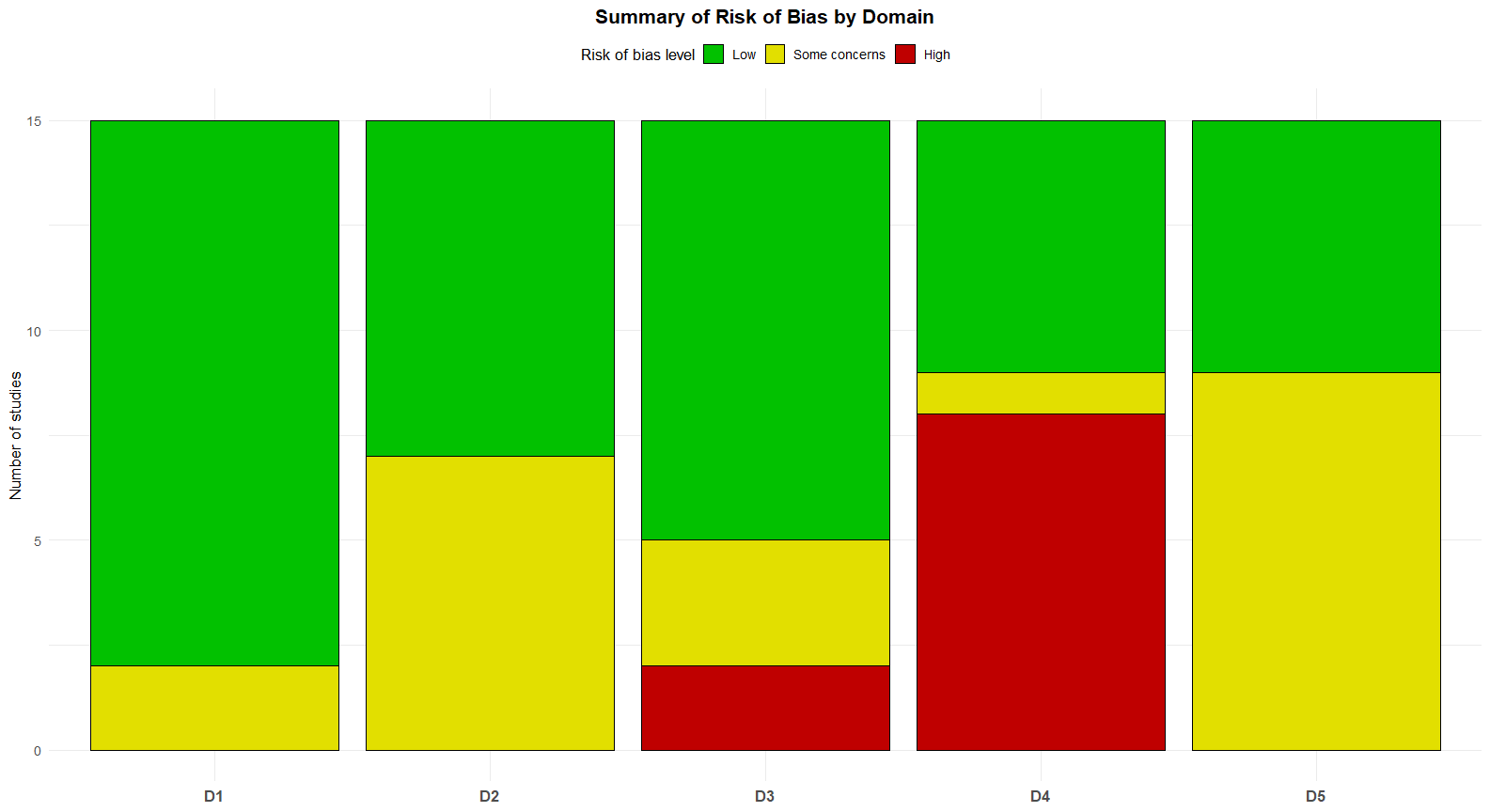

Supplement: Supplementary file 1 [file diagnostics-15-02684-s001.zip › Supplementary File S3.png]
